# ZFP36 stabilizes RIP1 via degradation of XIAP and cIAP2 thereby promoting ripoptosome assembly

**DOI:** 10.1186/s12885-015-1388-5

**Published:** 2015-05-06

**Authors:** Tommaso Selmi, Claudia Alecci, Miriam dell’ Aquila, Lucia Montorsi, Andrea Martello, Filippo Guizzetti, Nicola Volpi, Sandra Parenti, Sergio Ferrari, Paolo Salomoni, Alexis Grande, Tommaso Zanocco-Marani

**Affiliations:** 1 Department of Life Sciences, University of Modena and Reggio Emilia, Modena, 41125 Italy; 2 Samantha Dickson Brain Cancer Unit, UCL Cancer Institute, London WC1E 6BT, United Kingdom

**Keywords:** ZFP36, Ripoptosome, XIAP, cIAP2, RIP1, Glioma, Necroptosis

## Abstract

**Background:**

ZFP36 is an mRNA binding protein that exerts anti-tumor activity in glioblastoma by triggering cell death, associated to an increase in the stability of the kinase RIP1.

**Methods:**

We used cell death assays, size exclusion chromatography, Co-Immunoprecipitation, shRNA lentivectors and glioma neural stem cells to determine the effects of ZFP36 on the assembly of a death complex containing RIP1 and on the induction of necroptosis.

**Results:**

Here we demonstrate that ZFP36 promotes the assembly of the death complex called Ripoptosome and induces RIP1-dependent death. This involves the depletion of the ubiquitine ligases cIAP2 and XIAP and leads to the association of RIP1 to caspase-8 and FADD. Moreover, we show that ZFP36 controls RIP1 levels in glioma neural stem cell lines.

**Conclusions:**

We provide a molecular mechanism for the tumor suppressor role of ZFP36, and the first evidence for Ripoptosome assembly following ZFP36 expression. These findings suggest that ZFP36 plays an important role in RIP1-dependent cell death in conditions where IAPs are depleted.

**Electronic supplementary material:**

The online version of this article (doi:10.1186/s12885-015-1388-5) contains supplementary material, which is available to authorized users.

## Background

ZFP36 belongs to the TIS11/TTP protein family, a class of mRNA binding proteins carrying a conserved zinc finger domain, which binds to AU-rich elements in the 3′UTR of mRNAs. ZFP36 triggers mRNA decay and or inhibition of translation of its targets, resulting in negative regulation of gene expression [1]. A growing body of literature demonstrates the ability of ZFP36 to negatively regulate mRNAs coding for oncogenes in different cellular contexts thereby exerting anti-tumor activities [2-5]. Interestingly, it has been shown that ZFP36 sensitizes transformed cell lines to TNF alpha-induced cell death [6] and we have recently described the role of ZFP36 in promoting necroptosis in glioma cell lines [4].

Cells die by activating many forms of programmed cell death, such as apoptosis and necroptosis, which are characterized by the assembly of intracellular complexes that execute death sentences [7]. The Ripoptosome is a recently described death-inducing complex, whose assembly is triggered in responsive cancer cell lines by Etoposide or SMAC-mimetics [8, 9]. Once assembled, this complex signals both apoptotic and necroptotic cell death, independently of the activation of the TNF-receptor superfamily [8]. The Ripoptosome contains the core components RIP1, caspase-8 and FADD. It has been suggested that the stability of this complex is regulated by the E3 ubiquitine ligases XIAP, cIAP1 and cIAP2, which act by marking the core components for proteasome-mediated degradation [8]. Etoposide and Interferons are used to induce cell death dependent on RIP1 [8, 10] however, only few details are known regarding the intracellular mediators of the necroptotic effects of these drugs [11]. We previously demonstrated that ZFP36 acts as tumor suppressor in glioma cell lines by inducing necroptotic cell death and we speculated that RIP1 stabilization could be responsible for the execution of this death program [4]. Here we show that ZFP36 stabilizes RIP1, promotes the assembly of the Ripoptosome and induces RIP1-dependent cell death. The key molecular event underlining this process is the depletion of the IAP proteins XIAP and cIAP2, which we and others [4, 12] previously described as a target of ZFP36-mediated mRNA decay. Finally we demonstrate that loss of ZFP36 in glioma cancer stem cells leads to the down-regulation of RIP1, thus reinforcing the molecular link between ZFP36 and RIP1.

## Methods

### Cell cultures

Human embryonic kidney (HEK) 293 T cells were obtained from ATCC and cultured in IMDM medium (Euroclone) supplemented with 10% heat-inactivated fetal bovine serum (Biowhittaker), 1 mM L-glutamine and penicillin/streptomycin (100 μg/ml) (Euroclone). As described by Pollard et al. [13], G144 human glioma neural stem (GNS) cell lines were maintained onto laminin (10 mg/ml, Sigma) coated dishes in expansion media (RHA-B media from Stem Cell Technologies) supplemented with 20 ng/mL of EGF (recombinant mouse, PeproTech 315–09 20 ng/ml) and 20 ng/mL bFGF (recombinant human, PeproTech100-18B, 20 ng/ml). Medium was changed every 2 days, and cells were split 1:2 to 1:3 once culture became confluent.

### Chemicals and treatments

Where indicated, HEK293T were treated with 100 μM Etoposide (Invitrogen), 20 μM Z-VAD-FMK (Invitrogen), 50 μM Necrostatin-1 (Enzo Life Sciences) and DMSO (Sigma) served was administered as vehicle.

### Expression constructs

Full-length ZFP36 was cloned in the pCDNA3.1 vector (Invitrogen) as already described in reference 4. The pCI-IRES-HA-XIAP (Δ3′UTR XIAP) allows the expression of the XIAP coding sequence driven by its own IRES and was a gift from Dr Salomoni.

### Cell death assays

HEK293T were transfected using the Transit 2020 Reagent (Mirus Bio LLC) according to manufacturer’s instruction. In the co-transfection experiments, the same amount in μg for each plasmid was added to the transfection mix. 48 hours after transfection, cell death was assessed by Propidium Iodide (PI) or Annexin V/Propidium Idodide staining (AV/PI). For PI staining, cells were cultured in the presence of 0.5 μg/mL PI (BD PharMingenTM) for 15 minutes and imaged using a Leica 5515015 inverted microscope. AnnexinV/PI positivity was assessed by BD PharMingenTM kit (cat. Number 556547) at the cytofluorimeter (Coulter Epics XL MCL, Beckman Coulter Inc.). Trypan Blue positivity was used to count dead cells in Neubauer Chambers. HEK293 cells were gently detached from plates after short incubations (60″) in 1X Trypsin-EDTA solution (Life Technologies). Error bars represent mean and SEM calculated on a set of three to four independent experiments (*p < 0.05; **p < 0.01).

### Chromatographic separation

Cellular lysates were fractionated by gel-permeation size-exclusion chromatography on Sepharose 6B (Sigma-Aldrich, fractionation range for globular proteins 1 × 10^4^ ÷ 4 × 10^6^) column of 90 cm high and 1 cm of diameter. The column was eluted with 5% w/v sucrose, 0.1% w/v CHAPS, 20 mM HEPES, 5 mM DTT, and 50 mM NaCl, buffered at pH 7.0. Column flow was 1 mL/5 min and fractions volume was set at 2 mL. The column was calibrated with protein standards and blue dextran (Sigma-Aldrich). After elution, fractions were freeze-dried and further analyzed.

### Co-Immunoprecipitations, western blots and antibodies

For the Ripoptosome immunoprecipitation, cells were transfected with the ZFP36 expression vector or with the empty vector. 24 hours following transfection, Z-VAD-FMK and, where indicated, also Etoposide were added for further 24 hours. Cells were then lysed in 30 mM Tris (pH 7.4), 120 mM NaCl, 2 mM EDTA, 2 mM KCl, 10% glycerol, 0.2% NP-40, 1 mM sodium deoxycholate, 1 mM sodium orthovanadate and Complete Protease Inhibition Cocktail (Roche), and caspase-8 was immunoprecipitated with 2 mg of caspase-8 (1C12) monoclonal antibody (Cell Signaling, 9746) coupled to protein G agarose (KPL). The CO-IP negative control was immunoprecipitated using non-specific mouse IgG (Santa Cruz Biotechnology). Immunoprecipitates where resuspended in 2x Laemmli Buffer without SDS and heated at 100° for 5′ prior to loading onto SDS-polyacrylamide gels. Membranes were probed against RIP1 (BD Transduction Laboratories, #610458), caspase-8 (1C12) (Cell Signaling, 9746) and (BD Transduction Laboratories, #610399). IgG-HRP secondary antibodies were from Santa Cruz Biotechnology (sc-2031) and the Mouse TrueBlot (ebioscience).

Other antibodies used were: ZFP36 (Abcam, ab124024), TTP (H-120) (Santa Cruz biotecnology, sc-8458), XIAP (Cell Signaling, #2042), cIAP2 (H-85) sc-7944 (Santa Cruz Biotchnology), PARP-1 (F-2) sc-8007 (Santa Cruz biotecnology), MLKL M6697 Sigma-Aldrich), VINCULIN clone V284 (Merck Millipore). Expression of actin was analyzed with a mouse anti-human pan-actin MoAb (Sigma-Aldrich) to normalize protein samples.

### Lentivectors

To knockdown ZFP36 expression in human glioma neural stem cells, we used the commercially available pGIPZ-lentiviral shRNAmir vectors containing a hairpin sequence targeting ZFP36 (Open Biosystems). The hairpin sequence was as follows: CGACTTTATTTATTCTAATATTACATCTGTGGCTTCACTAATATTAGAATAAATAAAGTCG.

The shRNA-containing lentiviral vector (Open Biosystems) was cotransfected with lentiviral packaging constructs into HEK293T cells to produce shRNA-carrying lentivirus particles. Supernatants were collected at 48 h after transfection and viral particles were concentrated using PEG (System Biosciences). GNS cells were transduced (MOI = 10) for 1 h and subsequently supplemented with fresh media. After expansion, cells were sorted (MoFlo cell sorter, Beckman Coulter Inc.) based on GFP expression values, obtaining two homogeneous populations expressing either the (pGIPZ) scrambled vector, or pGIPZ shRNA ZFP36 (shZFP36). To perform over-expression experiments, the coding sequence of ZFP36 [4] was cloned in the pRRLSIN.cPPT.PGK-GFP.WPRE vector. The procedure for producing the lentiviral particles and infecting the cells was the same as described for the pGIPZ vectors. ZFP36 expressing cells were used for experiments 24 to 48 hours after infection.

## Results

### ZFP36-mediated RIP1 stabilization depends on IAPs degradation and correlates with increased cell death

**Figure 1:**
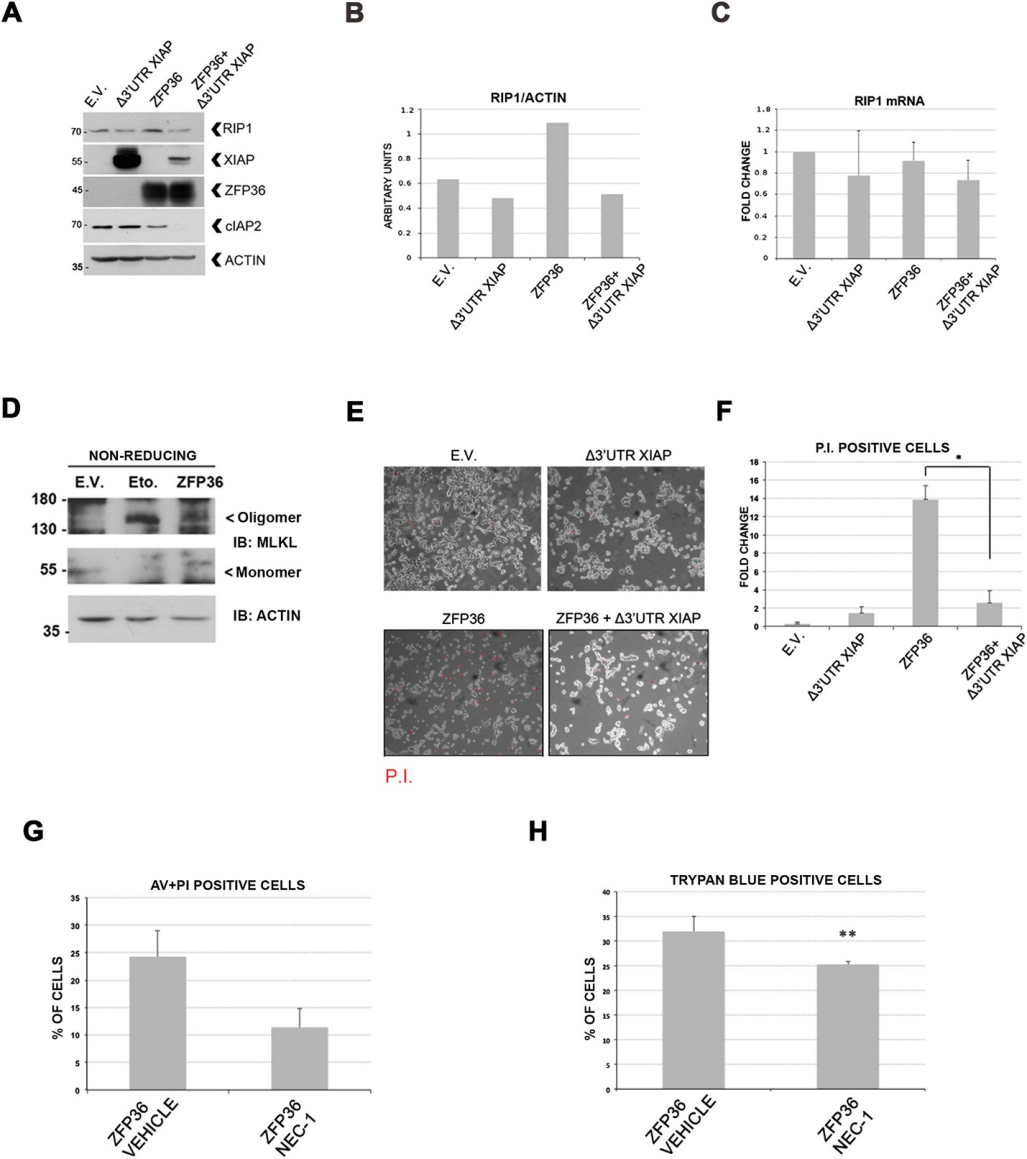
ZFP36 controls RIP1 stability by depleting IAPs and promotes RIP1-dependent cell death. **A)**. Transfection of HEK293T cells with a ZFP36 construct (ZFP36) correlates with the depletion of cIAP2 and with an increase in the stability of RIP1 at 48 hours. HEK293T cells transfected with a XIAP construct depleted of its own 3′UTR (Δ3′UTR XIAP) show a reduction in the levels of RIP1, even in the presence of ZFP36 (ZFP36 + Δ3′UTR XIAP). **B)** Image J software was used to normalize the levels of RIP1 over beta-ACTIN. **C)** Real Time PCR showing RIP1 mRNA levels following ZFP36 and Δ3′UTR XIAP over-expression. **D)** Both the transfection of ZFP36 and the treatment with Etoposide induce the formation of MLKL homo-oligomers and a decrease of MLKL monomers. E.V. cells were incubated with DMSO as this is the vehicle for Nec-1 administration. Non-reducing conditions were applied in order to preserve intact oligomers of MLKL. **E)** HEK293T transfected with the ZFP36 construct (ZFP36) show increased positivity to Propidium Iodide staining (P.I.), which is reduced in the presence of the Δ3′UTR XIAP construct (ZFP36+ Δ3′UTR XIAP). **F)** Percentage of P.I. positive cells obtained from 5 fields for each condition from experiment 1C. **G**-**H)** 48 hours treatment with 50 μM Necrostatin-1 inhibits ZFP36-dependent cell death (ZFP36 Nec-1) if compared to DMSO treated cells (ZFP36 vehicle), as assessed by cytofluorimetric analysis of Annexin V / P.I. or positivity to Trypan blue. The graphs in this figure represent the mean and SEM calculated on a set of three to four independent experiments (*p < 0.05; **p < 0.01).

Experimental evidences demonstrate that ZFP36 triggers the degradation of XIAP and cIAP2 mRNAs, which carry AU-rich elements in their 3′UTR [4, 12]. Figure 1A shows that in HEK293T cells, that endogenously express cIAP2 and lower levels of XIAP, ZFP36 over-expression determines the down-regulation of cIAP2, which correlates with an increase in RIP1 (Figure 1A, B). This confirms that loss of IAPs activity can stabilize RIP1 [8, 14] and suggests that ZFP36 might regulate RIP1 through IAPs degradation. Accordingly, the observed increase in RIP1 was abolished by the co-transfection of a XIAP construct lacking the 3′UTR, which cannot be degraded by ZFP36 (Δ3′UTR XIAP) (Figure 1A, B). This was further confirmed when a more physiological induction of ZFP36 was achieved by means of lentiviral infection (results are shown in additional file 1 A, B) in the supplementary material section. In order to verify whether the increase of RIP1 depends on protein stabilization rather than on increased mRNA expression, we analyzed by Real Time PCR the levels of RIP1 mRNA. No statistically significant changes in RIP1 mRNA levels were detected (Figure 1C), suggesting that RIP1 protein is stabilized following ZFP36 expression.

Mixed lineage kinase domain-like protein (MLKL) is currently the main confirmed effector of RIP kinases-dependent necropotsis [15]. Upon activation of necroptosis, the monomeric form of MLKL decreases, owing to the formation of homo-oligomers that relocate to the plasma and intracellular membranes, where they compromise membrane integrity, resulting in cell death [15]. As a result of ZFP36 overexpression, the monomeric form of MLKL decreases while an increase of the homo-oligomeric form is observed (Figure 1D).

As cells overexpressing ZFP36 show an increase in RIP1 and in the oligomerized MLKL, we checked the permeability of these cells to propidium iodide. As shown in Figure 1E-F, where graph F recaps the percentages observed in 1E, cells overexpressing ZFP36 are more permeable to propidium iodide than cells expressing ZFP36 and the Δ3′UTR XIAP, suggesting that they are less sensitive to ZFP36-induced cell death (ZFP36 + Δ3′UTR XIAP). To confirm that ZFP36-induced cell death is dependent on RIP1 we inhibited this kinase with its inhibitor Necrostatin-1 (Nec-1). Figure 1G-H shows that Nec-1 partly restrains ZFP36-dependent cell death in HEK293T cells, thus confirming that ZFP36 triggers RIP1-dependent cell death. Collectively, the data shown in Figure 1 demonstrate that the negative regulation exerted by ZFP36 over the XIAP and cIAP2 E3 ubiquitine ligases is a key mechanism controlling the stability of RIP1 and the sensitivity to necroptotic cell death.

### ZFP36-mediated RIP1 stabilization leads to Ripoptosome assembly

**Figure 2:**
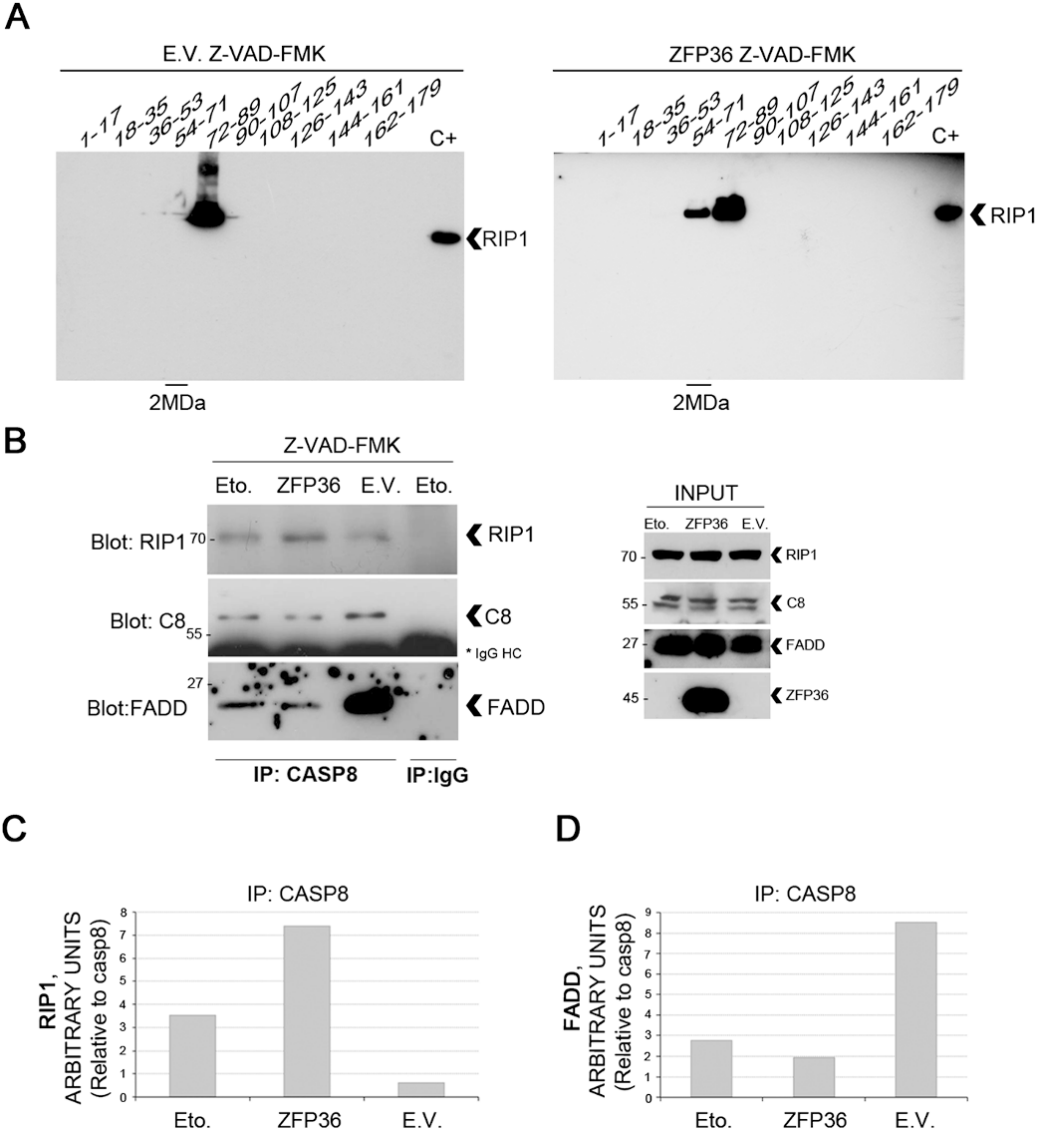
ZFP36 promotes RIP1 assembly in a high molecular weight complex containing caspase8 and FADD. **A**, **B)** Following 24 hours of empty vector/ZFP36 transfection, HEK293T cells were further treated for 24 hours in the presence of 20 μM Z-VAD-FMK, in order to block caspases’ activity and facilitate the recovery of the Ripoptosome, as described in Tenev et al. [8]. A positive control for Ripoptosome assembly was added in the co-immunoprecipitation experiments depicted in figure B, where HEK293T were treated for 24 hours with 100 μM Etoposide and 20 μM Z-VAD-FMK. **A)** 2 milligrams of lysates from either empty vector (E.V.) or ZFP36 transfected cells (ZFP36) were loaded on a Sepharose column and fractions were eluted at defined timing and then loaded on polyacrylamide gel to perform Western blotting. In the presence of ZFP36, RIP1 was detected in higher molecular weight fractions (54–71), while this was less evident in E.V. samples. A positive control for RIP1 was loaded for each sample series (C+). Molecular weight markers were eluted through the Sepharose column in order to identify the 2MDa fractions. **B)** Ripoptosome recovery was carried out by immunoprecipitating caspase8, while immunoprecipitation with nonspecific mouse IgG was performed on the Etoposide (Eto.) sample as a technical control for the procedure. Both in the presence of ZFP36 or Etoposide (Eto.) RIP1 was associated with caspase8, while this was less evident in the empty vector population (E.V.). Higher levels of FADD were associated to caspase8 in the empty vector population, as opposite to the ZFP36 and Etoposide (Eto.) samples. Input controls are represented on the right panel. **C**, **D)** Image J software provided a relative quantification of the levels of RIP1 and FADD associated to caspase8.

Based on these observations, we hypothesized that the increased availability of RIP1 could result in its loading into the 2MDa Ripoptosome. To assess this, we first confirmed the presence of RIP1 in high molecular weight complexes only in the presence of ZFP36 (Figure 2A) and then we co-immunoprecipitated the Ripoptosome components (Figure 2B-D). Our results demonstrate that the binding of RIP1 to caspase-8 is promoted by ZFP36 overexpression (Figure 2B, C), and that similar results are observed in response to Etoposide (Eto.), a Ripoptosome-stabilizing drug that acts through depleting IAPs [8]. When comparing empty vector (E.V.) to ZFP36-transfected cells (ZFP36), the amount of RIP1 bound to caspase-8 was strongly reduced (Figure 2C) concomitantly to increased association between caspase-8 and FADD (Figure 2D). This suggests that ZFP36 determines, by stabilizing RIP1, the aggregation of a complex which, according to published data [11, 16, 17], might unbalance cells towards necroptosis rather than apoptosis. Therefore, we conclude that ZFP36 controls RIP1 stability, promotes its assembly in a high molecular weight complex and enhances its association to caspase-8 and FADD.

### ZFP36-mediated RIP1 stabilization occurs in glioma neural stem cell lines

**Figure 3:**
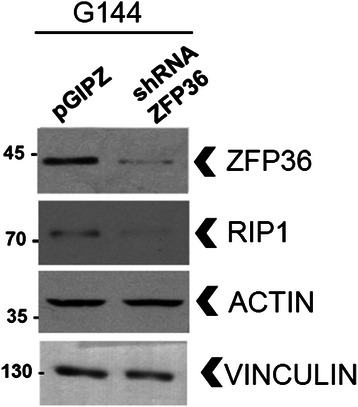
ZFP36 controls RIP1 stability in glioma neural stem cells. Human G144 glioma neural stem cells were grown in adhesion in proliferation medium and transduced with a short hairpin lentivirus directed against ZFP36 (shZFP36) or with a scrambled short hairpin sequence (pGIPZ) and sorted based on eGFP-positivity. Western blot analysis suggests that RIP1 stability is reduced in the absence of ZFP36 (shZFP36) when compared to G144 that retain ZFP36 expression (pGIPZ).

To test the relevance of our data, we assessed the ability of ZFP36 to control RIP1 stability in human glioma neural stem cell lines (GNS), as this population shows resistance to standard therapy and is believed to mediate tumor recurrence [13]. A panel of six different glioblastoma-derived GNS cell lines were screened for ZFP36 expression, which resulted to be very heterogeneous (data not shown). G144 GNS [18] express both ZFP36 and RIP1 when cultured in proliferation medium, and we detected a reduction in the total RIP1 levels (Figure 3) upon depletion of ZFP36, thus demonstrating that ZFP36 controls RIP1 stability in this cancer stem cell context.

## Discussion

RIP1 is a cytosolic molecule capable of mediating either NF-kB signalling in resting conditions or necroptotic and apoptotic cell death in response to stress [19]. The regulation of RIP1 activity is dependent on the IAP E3 ubiquitine ligases cIAP-1, cIAP-2 and XIAP, which constitutively ubiquitinate RIP1 in transformed cells, where K63 ubiquitination promotes the assembly of a pro-survival complex [19], while K48 might mediate RIP1 proteasomal degradation [8]. Anticancer drugs such as Etoposide and SMAC-mimetics induce loss of IAPs and promote Ripoptosome assembly, while some natural molecules such as Interferons, are able to promote RIP1-dependent necroptosis [11]. A recent report demonstrated that Etoposide induces ZFP36 expression [20] and, moreover, it is interesting to note that ZFP36 levels rise in response to the “breast cancer susceptibility protein” BRCA1 [21], which is implicated in the DNA damage response. Even though Interferons induce ZFP36 expression [22], it is still unknown whether ZFP36 is involved in mediating necroptosis in this setting.

Our data show that the ZFP36 mRNA binding protein regulates negatively the stability of XIAP in glioma cancer cells [4] and here we demonstrate in HEK293T cells that ZFP36-dependent destabilization of cIAP2 and XIAP leads to an increase in RIP1 levels and favors RIP1 association to caspase-8 and FADD, while inducing cell death. These observations are consistent with described NF-kB inhibitory activity of ZFP36 [23] and with reports of synergism between ZFP36 and TNF alpha in inducing cell death in HEK293 cells [6]. Since our approach relies on ZFP36 restoration through transfection and is dependent on E3 ubiquitine ligases depletion, we identify the described molecular complex as the Ripoptosome. However, we cannot exclude that ZFP36 could mediate the association of RIP1, caspase-8 and FADD also in response to death receptor signaling, as ZFP36 is induced and post-translationally regulated in response to TNF receptor activation [24, 25].

The observed control of ZFP36 over RIP1 stability in GNS cell lines further supports the molecular mechanism here described and suggests that GNS cell lines that loose ZFP36 expression may be less sensitive to necroptotic-inducing treatments. Taken together, our results demonstrate a novel role for ZFP36 in regulating Ripoptosome assembly and RIP1-dependent necroptosis through IAP depletion.

## Additional file

Additional file 1: **ZFP36 controls RIP1 stability by regulating IAPs.** The expression of ZFP36 in HEK293 (pRRL-ZFP36) with a lentiviral vector increases the levels of RIP1, when compared to the empty pRRL vector (pRRL). This effect is restrained by the co-transfection of a XIAP expression construct depleted of its own 3′UTR (pRRL-ZFP36 + D 3′UTR XIAP).

## Abbreviations

ZFP36: Zinc finger protein 36 homolog
E.V: Empty vector
Eto: Etoposide
Nec-1: Necrostatin
IAPs: Inhibitor of Apoptosis Proteins
GNS: Glioma neural stem
TTP: Tristetraprolin
AU-rich: Adenine Uridine-rich
TNF alpha: Tumor necrosis factor alpha
RIP1: Receptor interacting serine/threonine protein kinase 1
FADD: Fas associated-protein with death domain
TRAF2: TNF receptor-associated factor 2
cIAP2: Cellular inhibitor of apoptosis 2
XIAP: X-linked inhibitor of apoptosis
3′UTR: 3′untranslated region
P.I: Propidium Iodide
MDa: Mega Dalton
DMSO: Dimethyl sulfoxide
MLKL: Mixed lineage kinase domain-like
