# Supplementary material for: ZFP36 stabilizes RIP1 via degradation of XIAP and cIAP2 thereby promoting ripoptosome assembly
Source: BMC Cancer. 2015 May 6;15:357. doi: 10.1186/s12885-015-1388-5 (PMC4424499; doi:10.1186/s12885-015-1388-5)
Supplement: Additional file 1: — ZFP36 controls RIP1 stability by regulating IAPs. The expression of ZFP36 in HEK293 (pRRL-ZFP36) with a lentiviral vector increases the levels of RIP1, when compared to the empty pRRL vector (pRRL). This effect is restrained by the co-transfection of a XIAP expression construct depleted of its own 3′UTR (pRRL-ZFP36 + D 3′UTR XIAP). [file 12885_2015_1388_MOESM1_ESM.pdf]

Additional File 1

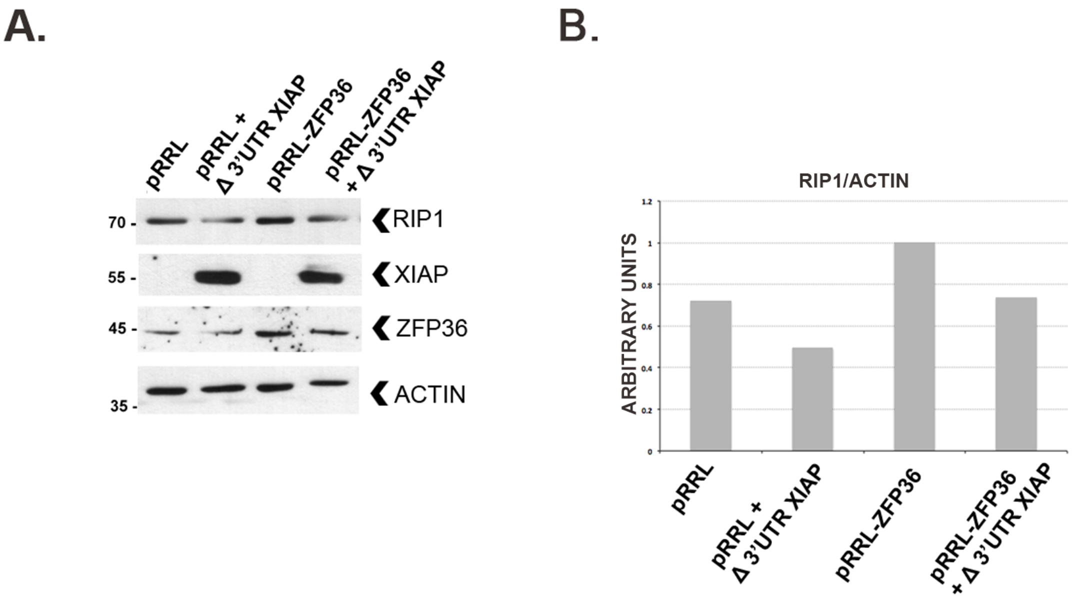

ZFP36 controls RIP1 stability by regulating IAPs

A. RIP1 levels increase following the infection of HEK293 with a lentiviral vector coding for ZFP36 (pRRL-ZFP36). This effect is restrained by the co-transfection of a XIAP construct lacking the 3' UTR (pRRL-ZFP36+Δ3'-UTR XIAP). **B.** Levels of RIP1 normalized over actin for the experiment shown in A.
